# Vitamin D Binding Protein (VDBP) and Its Gene Polymorphisms—The Risk of Malignant Tumors and Other Diseases

**DOI:** 10.3390/ijms21217822

**Published:** 2020-10-22

**Authors:** Dominika Rozmus, Alicja Ciesielska, Janusz Płomiński, Roman Grzybowski, Ewa Fiedorowicz, Natalia Kordulewska, Huub Savelkoul, Elżbieta Kostyra, Anna Cieślińska

**Affiliations:** 1 Faculty of Biology and Biotechnology, University of Warmia and Mazury, 10-719 Olsztyn, Poland; dominika.rozmus@uwm.edu.pl (D.R.); alicja.staruch@wp.pl (A.C.); ewa.kuzbida@uwm.edu.pl (E.F.); natalia.smulska@uwm.edu.pl (N.K.); ezlbieta.kostyra@uwm.edu.pl (E.K.); 2 Clinical Department of Trauma-Orthopedic Surgery and Spine Surgery of the Provincial Specialist Hospital in Olsztyn, 10-561 Olsztyn, Poland; plominsky@poczta.onet.pl (J.P.); romek.grzybowski@wp.pl (R.G.); 3 Department and Clinic of Orthopaedics and Traumatology, Collegium Medicum, University of Warmia and Mazury, 10-719 Olsztyn, Poland; 4 Cell Biology and Immunology Group, Department of Animal Sciences, Wageningen University and Research, 6700 AG Wageningen, The Netherlands; huub.savelkoul@wur.nl

**Keywords:** vitamin D binding protein, vitamin D, VDBP, SNP, single nucleotide polymorphism, cancer, [25(OH)D]

## Abstract

Vitamin D is an important component of the endocrine system that controls calcium homeostasis and bone mineralization. Because of the very short half-life of free serum vitamin D it is stabilized and transported to target tissues by being bound to the vitamin D binding protein (VDBP). The most common polymorphisms: rs4588 and rs7041 in the vitamin D binding protein gene may correlate with differences in vitamin D status in the serum. This review presents data that relate to the presence of genetic variants in the VDBP gene in correlation with certain diseases, mostly concerning cancers (breast, prostate, pancreatic, lung, colorectal, basal cell carcinoma cancer and cutaneous melanoma) or other related diseases (thyroid autoimmunity disorders, obesity, diabetes mellitus, bone metabolism, rheumatoid arthritis, ankylosing spondylitis, asthma, chronic obstructive pulmonary disease, tuberculosis and coronary artery diseases).

## 1. Introduction

Vitamin D plays a crucial role in the endocrine system which controls calcium homeostasis in the whole body [1] and along with parathyroid hormone—bone mineralization [2]. A proper 25-hydroxyvitamin vitamin D [25(OH)D] status helps to maintain a healthy body weight and prevents obesity [3]. Vitamin D takes part in innate and adaptive immunity and is involved in the detoxification of bile acids [4], it plays important role in intestinal homeostasis by limiting microbiota entrance into interstitium and regulates the immune system by favoring formation of Treg cells and suppressing Th1/Th17 cells [5]. Vitamin D receptor (VDR), enzymes and metabolites have different expression levels in various types of immune cells such as lymphocytes, monocytes, macrophages and dendritic cells [6,7].

Vitamin D is classified as a steroid hormone with wide regulatory effects. It affects regulation of the expression of many different genes that are involved in the differentiation, activation and proliferation of many cell types. The active form of vitamin D may inhibit the expression of interleukin 2 and interferon γ and affect the differentiation and maintenance of the balance of regulatory cells [8]. The action of vitamin D in cells requires the presence of nuclear and cytosolic vitamin D receptors (VDR). VDR binds the vitamin D active metabolite: 1α,25-dihydroxyvitamin D3. VDR is expressed in nerve cells, glial cells and cells of the immune system, such as monocytes, macrophages and activated T and B lymphocytes, as well as in cancer cells (colon cancer) and liver stellate cells. The presence of these receptors allows for the regulation of gene expression involved in organ development, cell cycle control, calcium and phosphate homeostasis in bone metabolism and xenobiotic detoxification. Research literature suggests that up to 500–1000 genes can be modulated by VDR ligands [9].

**Figure 1:**
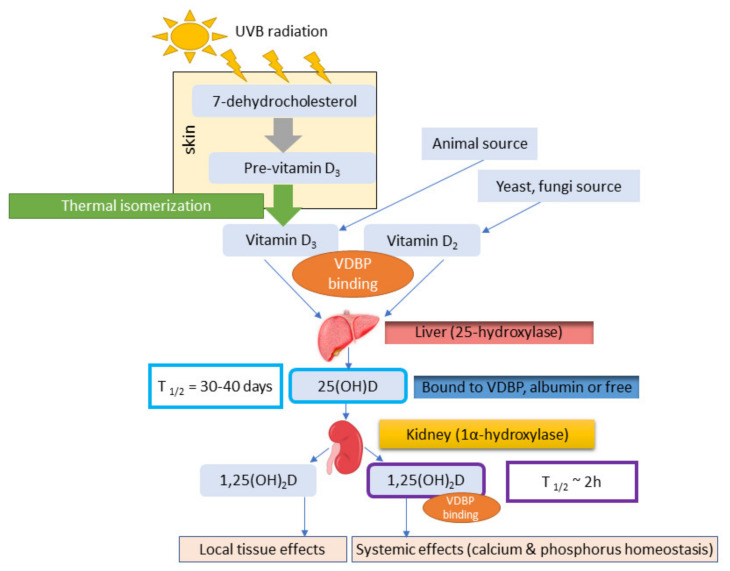
Vitamin D synthesis pathway (based on Bikle 2014 [14])**.**

Human can synthesize vitamin D under UVB radiation exposure or obtain it through oral intake from diet and supplements [10]. There are two major forms of the vitamin D, D3 (cholecalciferol), which is an animal form and D2 (ergocarciferol), which is present in plants but mostly in fungi and yeasts [11,12,13]. The diet or ingestion of supplements can be an important source for vitamin D in humans. Vitamin D3 can be synthesized endogenously and it is produced by the skin upon UVB exposure [13]. The photoproduction of vitamin D in the skin begins with the synthesis of 7-dehydrocholesterol (7-DHC) (Figure 1). 7-dehydroholesterol (7-DHC) is built into the cell membranes of the epidermis and dermis. When exposed to solar UVB, 7-DHC is converted into pre-vitamin D3, which is isomerized to cholecalciferol in the cell membrane by a subsequent thermal isomerizatoin [10]. The cholecalciferol produced is removed to the extracellular space and reaches the capillary in the dermis by diffusion. Cholecalciferol is transported to the liver by the vitamin D binding protein (VDBP) and then it is converted to 25-hydroxyvitamin D [25(OH)D3, calcidiol]. 25(OH)D3 is the major circulating metabolite of vitamin D, which is hydroxylated in the kidney by 1-alpha-hydroxylase to its active form: 1,25-dihydroxyvitamin D [1,25(OH)_2_D3, calcitriol] [14]. Conversion from calcidiol to calcitriol also occurs in nonrenal tissues and cells (NRTCs): skin, parathyroid glands, bone cells, cardiovascular and immune cells [15].

Low vitamin D status have been linked to bone mineralization defects, as well as common chronic, autoimmune and infectious diseases including cancer, cardiovascular disease, diabetes, multiple sclerosis, rheumatoid arthritis and tuberculosis [16,17,18,19,20,21,22,23,24,25,26,27,28,29]. Other studies do not support the theory of protective effects of vitamin D in cancer or cardiovascular disease: taking up to 2000 IU of vitamin D per day by men and women had no reduction in invasive cancer or major cardiovascular cases compared to placebo group. Supplementation of vitamin D with 2000 IU dose was not associated with lower risk of having invasive cancer or composite of major cardiovascular event such as myocardial infarction, stroke or death due to cardiovascular causes. However, hazard ratio of incidence to any invasive cancer was lower among participants with BMI < 25 [30]. Meta-analysis of randomized controlled trial (RCTs) proved that vitamin D supplementation reduces total cancer mortality but do not reduce total cancer incidence [31]. Study of Barbarawi’s et al. including RCTs (83,291 patients with 41,622 placebo ones) showed that vitamin D supplementation was not associated with reduced major cardiovascular events [32].

Our review was conducted by searching phrases: “rs7041/rs4855 + disease”; vitamin d binding protein polymorphism; vitamin d binding protein polymorphism + disease; Gc globulin polymorphism + disease, rs7041/rs4855 + cancers; rs7041/rs4855 + chronic diseases; VDBP + disease; DBP + disease; 25-hydroxyvitamin D cancers; 25-hydroxyvitamin D + diseases; VDBP polymorphism disease; VDBP SNP disease. Google scholar and PubMed databases were used.

## 2. Vitamin D and Gene Regulation

When 1,25(OH)2D3 is attached to the nuclear and cytosolic vitamin D receptors (VDR), it is able to regulate many genes, including the genes encoding osteocalcin, osteopontin and Cyp24a1, as well as Vdr, Cbs, Tnfsf11, c-FOS, Spp1, Runx2, Cdon, Mmp13, Col2a, Trpv6, S100g and others [14,33]. The active transcription unit in 1,25(OH)2D3-mediated gene regulation is the VDR/RXR heterodimer. There are approximately 2000–8000 binding sites in human genome for VDR and the distal-binding site is distributed across the genome in cis-regulatory modules (enhancers or CRM). The number and location of those sites depend on the cell type but they can be found near promoters, mainly within introns or in distal intergenic regions and also in clusters of elements. VDR/RXR-binding site sequence (vitamin D responsive element, VDRE) can be inducted by classic hexameric half-sites (AGGTCA) separated by three base pairs. CRMs contain binding sites for many types of transcription factors [34,35,36]. Comparison of the VDR/RXR cistrome in early precursors and late mineralizing osteoblasts showed a reduction in the number of VDR binding sites in more mature cells and a reduction in their sites, especially near genes that were no longer responsive to 1,25(OH)2D3 [36].

### 2.1. Vitamin D3 and D2

**Figure 2:**
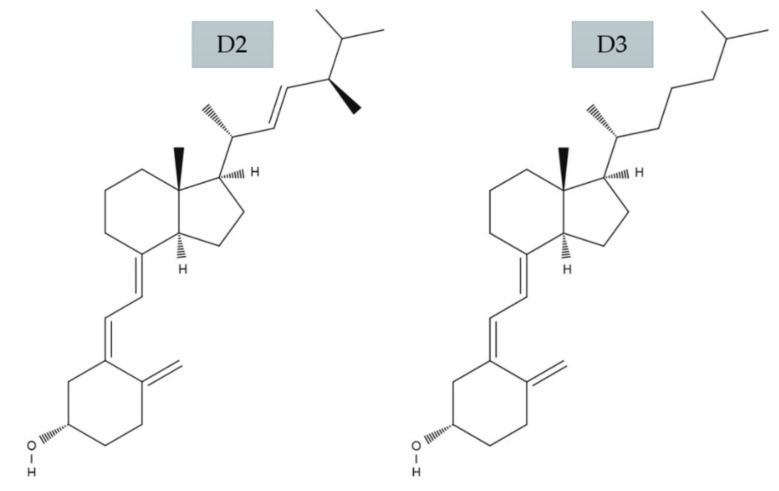
Molecular structure of vitamin D2 and D3 (based on Holick 2018 [38])**.**

Dietary sources of vitamin D include cholecalciferol (vitamin D3) and ergocalciferol (vitamin D2) [4]. Vitamin D3 is naturally produced in the skin of animals, including humans and vitamin D2 is produced in fungi, yeast and plants after exposure to sunlight and UVB radiation [19,37] (Figure 2).

Vitamin D2 (C28H44O) differs from D3 (C27H44O) by a side chain attached to the secosteroid backbone that contains a group set at carbon 24 and a double bond between carbon atoms 22 and 23. This difference means that the curb mass of D2 (396.65 g/moL) is 3.1% richer than vitamin D3 (384.64 g/moL) [39]. There are many studies proving that vitamin D3 has a much stronger effect than vitamin D2 by measuring 25(OH)D concentrations in both serum and adipose tissue [20,40,41,42,43].

### 2.2. Role of VDBP

Vitamin D binding protein is a serum α2-globulin of 52–59 kDa molecular weight. Originally it was called “group-specific component”—Gc [44]. The encoding VDBP gene (35kb DNA with 13 exons, 12 introns) is located on the long arm of the chromosome 4 (4q12-q13). VDBP belongs to the albumin superfamily of binding proteins (albumin, alfa-fetoprotein, alfa-albumin/afamin). They are expressed in the liver [45,46,47].

VDBP has many biological functions: binding and transporting of all vitamin D metabolites: (25OHD lactone > 25OHD = 24,25(OH)2D = 25,26(OH)2D > 1,25(OH)2D > vitamin D and vitamin D3 metabolites > vitamin D2 metabolites), binding of actin monomers and playing a crucial role in depolymerization of extracellular actin filaments [48], binding of fatty acids [34] binding to membranes proteoglycans of leucocytes and activation of complement C5 system [35].

### 2.3. VDBP Gene Family Polymorphism

The VDBP amino acid sequence consists of 458 amino acids arranged over three domains. The 16 N-terminal amino acids act as a signal peptide. VDBP can be glycosylated to a varying degree depending on the genotype [49]. The two SNPs—rs7041 and rs4588—in exon 11, located in domain III, correspond to the three major VDBP types (VDBP1F, VDBP1S and VDBP2). Apart from them, over 120 other types are distinguished [50].

These phenotypic alleles differ by a single-nucleotide polymorphisms (SNP) in exon 11: rs7041 and rs4588 and also by a glycosylation pattern [51]. An amino acid substitution difference of GC1F and GC1S occurs in position 416, where aspartic acid is substituted by glutaminic acid. The difference between GC1F and GC2 is a single amino acid substitution from threonine to a lysine (ACG to AAG) [52,53]. It is known that GC1 and GC2 are marked by an O-glycolisation [52], while GC1F and GC1S are modified with a N-acetyl-D-glucosamine core [54].

Alleles frequency has been examined and less predominance is shown for GC2 allele in comparison to GC1 allele and also GC1F allele has a lower frequency for white skin population, while the highest frequency was found for the GC1S allele. GC1F frequency for dark skin populations can explain its efficient transport of vitamin D metabolites. Skin pigmentation and intensity of sunlight exposure might thereby correlate with VDBP frequencies of human populations. It is known that more pigmented or keratinized skin types have a lower UV light penetration level and the higher risk of susceptibility to rickets [55].

Other studies have corelated SNPs of the VDBP gene to status of circulating 25(OH)D. An inverse correlation was found in Germany, where the number of rarer alleles of VDBP gene SNPs [rs4588 (C>A), rs2282679 (A>C) or rs1155563 (T>C)] was associated with higher hydroxyvitamin D status, mostly during summer. That can be explained by the hypothesis that genetic differences have an impact on interindividual variations of vitamin D status and responses to sun exposure—UVB radiation—which is a season-dependent environmental factor [56,57]. Another study shows that status of 25(OH)D were strongly related to VDBP polymorphisms in both SNPs: rs7041 and rs4588, especially when there is a high concentration of vitamin D in need of transportation [57].

Table 1 presents the characteristics of VDBP main polymorphism types.

## 3. Association of VDBP with Human Diseases

### 3.1. Cancers

#### 3.1.1. Breast Cancer

Abbas et al. in 2008 examined vitamin D pathway gene polymorphisms and association of VDBP with postmenopausal breast cancer risk. 25(OH)D as a biomarker for vitamin D concentration in humans was also a part of the study. 25(OH)D and VDBP polymorphisms have an impact on the decreasing risk of postmenopausal breast cancer. Also, there was no association between 25(OH)D status and VDBP type. Decreased risk of postmenopausal breast cancer was noted for GC2 allele in VDBP. The mechanism underlying this process is probably an anticancerogenic effect associated with Gc potential to convert Gc to GcMAF—a macrophage activator [51,54]. VDBP is required for proper macrophage activation. When a neoplastic tissue is present, Gc-globulin is hydrolyzed and activated with the sialidase of T lymphocytes and β-galactosidase to yield a highly potent MAF. Conversion of Gc-globulin to Gc-MAF makes macrophages activated to phagocytosis of cancer cells [58,59]. Recent studies show that immunotherapy with GcMAF has promising results in breast cancer therapy because of the potential of macrophages to infiltrate tumors and their crucial role in antitumor immunity. Macrophages activated by GcMAF can recognize, internalize and eliminate not only cancerous cells but also bacteria [60]. Another study shows association between VDBP rs7041 and breast cancer [61]. It is important to mention that a recent study showed that higher 25(OH)D concentrations (≥60 ng/mL) had the most protective effect in decreasing the breast cancer risk for about 80% [62]. Among women, plasma concentrations of ≥40 ng/mL 25(OH)D were associated with a substantial reduction on the risk of all invasive cancers combined [63].

#### 3.1.2. Prostate Cancer

Weinsten et al., (2012) reported a significant association between men with higher 25(OH)D status and the risk of prostate cancer. Status of 25(OH)D free fraction were also a part of the study, as well as VDBP serum concentration. Higher VDBP was related to decreased risk among men with lower 25(OH)D concentration. Speculation about the underlying mechanism was directed at higher levels of VDBP that may bind more vitamin D by prostatic epithelium. Prostate tissue was shown to express megalin that has functions in absorption of many molecules, also hormones as SHGB. SHGB-bound testosterone was associated with prostate tumor progression. 25(OH)D and testosterone status are associated [64]. However, another study does not show any significant correlation with protective association between higher vitamin D status and lower risk of prostate cancer. Moreover, correlation between rs2282679 and risk of prostate cancer was rejected [65]. More recent meta-analyses are contradictory: the highest concentration of 25(OH)D is associated with an elevated risk of prostate cancer [66], while the vitamin D intake can decrease the risk of PC [67]. Moreover, vitamin D has influence on phosphorus homeostasis and phosphorus is association with risk of lethal and high-grade prostate cancer. Twenty four-year follow-up study suggested that calcium intake >2000 mg per day were associated with higher risk of total prostate cancer and phosphorus intake was associated with higher risk of total, lethal and high-grade cancers [68]. Calcium intake was associated with aggressive prostate cancer and vitamin D intake had inverse effect. However, those associations are race-, ethnicity- and BMI-dependent [69].

#### 3.1.3. Pancreatic Cancer

The interaction between VDBP and 25(OH)D was examined by Weinsten et al., (2012). The study shows association between higher concentration of circulating 25(OH)D and raised pancreatic cancer risk. Men with higher concentrations of 25(OH)D and VDBP in serum showed elevated risk of pancreatic cancer [64]. Another study found no relation of VDBP to pancreatic cancer [70]. Meta-analysis of Gc polymorphisms rs2282679, rs7041 and rs4588 found no significant correlation with pancreas cancer [71].

#### 3.1.4. Lung and Colorectal Cancer

A study showed that a low VDBP level in serum might be a predictor of subsequent death from lung cancer because the expression of VDBP gene is rather low or absent in lung cancer tissue [72]. The underlying mechanism might be the Gc potential to be converted into GcMAF (described in Breast Cancer subsection) and its subsequent antitumor effect. A second mechanism might be the potential of macrophages to convert 25OHD3 to 1,25(OH)2D3. 25OHD3 can be converted into 1,25(OH)2D3 by macrophages or other cells that express 1α-hydroxylase [72,73]. A study that proved correlation of VDBP polymorphisms with lung cancer was conducted among Thai patients. The TT-CA combination had protective association with lung cancer. The same study proved also that rs7041 (TG/GC) polymorphism was associated with colorectal cancer among 60 years and older patients. Rs4588 (CA/AA) polymorphism was associated with colorectal cancer among 60 years old and younger man [74]. Another study showed a significant correlation between rs7041 in the GC gene and a reduction in Non-Small Cell Lung Cancer risk [75]. Carrying both polymorphisms: rs7041 polymorphism in Gc and CYP2R1/rs10741657 (enzyme for 25-hydroxylase that converts vitamin D to 1,25(OH)2D3) decreases the risk of colorectal cancer about 9–12% [76].

#### 3.1.5. Basal Cell Carcinoma

Basal cell carcinoma is the most common cancer in Caucasians but there was no significant correlation with susceptibility to one or multiple BCCs in general. At the same time, among 6 patients GC1F was noted to have a decreased risk of developing the first BCC, while among other patients with heterozygous GC1F genotype, there was a higher chance for a first BCC development. Although, Rs7041 and rs4588 may thus be associated with BCC development among younger patients but those speculations required more studies at the time [77].

#### 3.1.6. Cutaneous Melanoma

Cutaneous Melanoma is caused by a transformation of melanocyte and pigment producing cells. The response on exposure to UV induces the production of melanin by these cells. The association between VDBP rs12512631 and the risk of cutaneous melanoma was shown in a study that was conducted among a Spanish population, as the exposure to the sun is higher than in the other European countries, especially northern ones. This association was explained only by the fact that VDBP variants can modulate this protein expression besides its activity which may affect vitamin synthesis and its distribution [78]. A meta-analysis showed that the VDBP rs12512631 C allele was significantly associated with survival of CM patients. The explanation that was provided suggested that rs12512631 may affect the corresponding gene’s function or the vitamin D plasma status and subsequently modulated the risk of death [79]. Schäfer et al., in 2012 showed the analysis of VDBP rs1155563 and rs7041 for their association with melanoma risk and prognosis. However, none of these two polymorphisms was associated with melanoma risk [80].

### 3.2. Other Important Diseases

#### 3.2.1. Diabetes Mellitus

The study of Hirai et al. in 2000 showed that a polymorphism of VDBP was associated with insulin resistance in Japanese with normal glucose tolerance. That may lead to type 2 diabetes development. This insulin resistance was different among the VDBP genotype since GC1S-2 and 1S-1S genotypes had higher fasting plasma concentration compared to 1F-1F [81,82]. Also another study on Pakistani population showed the association of VDBP to type-2 diabetic patients. In addition, vitamin D deficiency was correlated with an increased incidence of diabetes [83]. A recent study shows that rs7041 (Glu/Glu-416) and rs4588 (Lys/Lys-420) variants of VDBP were higher in type 2 diabetic comparing to control group. A higher risk of developing type 2 diabetes was associated among patients with Glu/Glu and Lys/Lys genotypes at codon 416 and 420, respectively [84]. Another recent study on the group of 2423 patients shows no connection between vitamin D insufficiency and higher risk of type 2 diabetes [85].

#### 3.2.2. Thyroid Autoimmunity Disorders

As vitamin D serum status among patients with Graves’ disease were lower compared to patients with nonautoimmune hyperthyroidism, an influence of VDBP polymorphism on the disease risk was suggested. Indeed, intron 8 (TAAA)n-Alu repeat polymorphism correlates with Graves’ disease (n = 561) but there is no association with Hashimoto’s thyroiditis [86]. Another study showed that VDBP polymorphisms may contribute to development of autoimmune diseases and they are associated with susceptibility to Grave disease in Polish population. Variations of VDBP might have influence on many immune functions [87].

#### 3.2.3. Obesity

VDBP has its function in the vitamin D and glucose metabolism and it is also associated with insulin resistance. A study showed a correlation between different SNPs and obesity among women, especially with respect to the percentage of fat mass (PFM) [88]. Insufficiency of vitamin D among obese people might thus be the result of decreased bioavailability of vitamin D3 and this bioavailability might be decreased because of vitamin D3 deposition in body fat mass compartments [89].

#### 3.2.4. Bone Metabolism

Powe et al. in 2011 showed that total 25(OH)D concentration status was positively correlated with VDBP levels but levels of VDBP-25(OH)D had no association with BMD (bone mineral density) [90]. This study was criticized and the apparent paradox was explained. Powe’s estimations were found incorrect because of the indirect method used for estimating of free 25(OH)D [91]. A recent study proved that low serum VDBP levels correlate with low BMD and that is why VDBP could have a potential as a non-invasive biomarker for early osteoporosis detection. The study focused on a group of postmenopausal women aged ≥45 years old but also had limitations as low BMD is not a disease marker but it reflects the bone status. Defining a sensitive and predictive biomarker for osteoporosis detection would thus be beneficial for such patients [92]. VDBP is the key for regulating calcium homeostasis. A study showed 13 SNPs among postmenopausal Japanese women and analysis suggested that multiple of the VDBP SNPs might increase the risk of osteoporosis in postmenopausal women [93]. VDBP is not the only one factor that can have influence on bone mineral density but rs7041 had significant association with BMD-L4 and a higher frequency of osteoporosis [94].

#### 3.2.5. Rheumatoid Arthritis

A study by Yan et al. showed a significant correlation between RA and rs2282679 SNP [95]. Rs4588 and rs7041 are the main genetic factors that contribute to variations in 25(OH)D status as well as variations in VDBP levels [96]. This genetic effect might be connected to a novel pathogenic pathway that vitamin D takes part in [95]. 1,25(OH)2D3 may have inhibitory effect on osteoclasts formation that is induced by IL-22 in RA [97,98]. Substitution of amino acids in the different isoforms of VDBP, like 1S and 2 isoforms, differ from the 1F isoform by post-translational glycosylation. The deglycosylated form of VDBP can promote activation of macrophages and osteoclasts [99,100]. The differentiation of osteoclasts is induced by macrophage colony-stimulating factor (M-CSF) and the receptor activator of nuclear factor- κB ligand (RANKL). The active form of 1,25(OH)2D3 binds to the VDBP and is transported to target organ or tissue by the blood circulation. Active 1,25(OH)2D3 binds to VDR and by enhancing RANKL secretion, it regulates the activity of osteoblasts [100].

#### 3.2.6. Ankylosing Spondylitis

The study of Jung et al. showed that among VDBP variations (rs4752, rs222016, rs222020, rs3733359) there is an association with the development of peripheral arthritis or uveitis. The haplotype (AGGA) was protective from peripheral arthritis development, while the haplotype (GAAG) lowers the risk of uveitis. VDBP polymorphisms are associated with ankylosing spondylitis development that affects the axial spine, sacroiliac joints leading to bone formation and ankylosis [48].

#### 3.2.7. Asthma

A study among Chinese population showed two common polymorphisms: rs7041 and rs4588. Further analysis showed that Gc2 is strongly associated with the risk of asthma but not with the plasma concentration of 25(OH)D among asthma patients. Gc1 might actually confer a protective effect. VDBP enhances the chemotactic activity of monocytes and neutrophils modulates Th2-mediated inflammation and influences the susceptibility to asthma [101,102]. A recent study among childhood Chinese patients showed that rs7041 and rs4588 Gc polymorphisms are significantly associated with lower risk of bronchial asthma [103]. Another study from Egyptian children and adolescents showed that rs4588 CA and AA genotypes were protective, while rs7041 GG genotype had significantly higher frequency among patients [104]. Among Kurdish population rs7041 GG genotype was corelated with increased risk of asthma progression [105].

#### 3.2.8. Chronic Obstructive Pulmonary Disease (COPD)

In Icelandic COPD patients the association of Gc genotypes were examined. Results showed that Gc1F and Gc2 genotype have an effect on sputum production among patients and increase risk of developing COPD [106]. VDBP may be mediated by affecting on macrophage activation. Macrophages accumulate in the COPD patients lungs and can release neutrophil chemoattractants in case of activation [107]. Among Korean patients the GC2 variant was a significant risk factor for vitamin D deficiency which is mostly low among COPD patients and 1F-1S genotype was protective factor against deficiency of vitamin D [108]. High frequencies of the haplotypes in rs7041 and rs4588—GC1S/1S. High sputum VDBP levels in COPD stage I and II were observed only among GC1S/1S genotype. The results of this study might contribute to further exploring the use of VDBP as a COPD biomarker [109].

#### 3.2.9. Tuberculosis

Vitamin D deficiency has already been associated with susceptibility to active tuberculosis—one of the leading cause of death in the world. Among Gujarati Asians, Gc2 genotype was associated with higher susceptibility to active tuberculosis but at the same time there was no such association among Rio and Cape Town population. This may be explained by the increased sun exposure in the Rio and Cape Town populations. Moreover, the number of Gc2 allele carriers among the Gujarati Asians is small, so this study might not detect fully correct associations [107,110]. Among Taiwanese population there was a strong correlation between GC1F and tuberculosis [111]. Another study showed no significant correlation between rs7041 and rs4588 polymorphism and susceptibility to tuberculosis [112].

#### 3.2.10. Coronary Artery Diseases (CAD)

The study of Kiani’s et al. in 2019 showed the associations of SNPs of VDR (rs1544410G>A) and those in VDBP (rs7041 T>G) as examined among a population of West Iran. Rs1544410 G>A was more frequent among CAD patients and was a strong risk factor for CAD. Rs7041 T>G gene polymorphism has a strong protective role against AMVC (Aortic and Mitral Valves Calcification) among this population in Iran. Also, a strong association of vitamin D deficiency, lipid profile and VDR rs1544410G>A and VDBP rs7041 T>G gene polymorphisms might be the reason of an increased CAD risk among these patients [113]. Another study showed that both rs7041 and rs4588 polymorphisms are not associated to the prevalence and extent of CAD.

Table 2 summarizes the knowledge on the VDBP influence on diseases epidemiology.

## 4. Conclusions

VDBP and vitamin D have a profound impact on humans’ health. Polymorphisms in VDBP gene can be a significant risk factor in many diseases, including cancers. SNPs genotyping is an easy procedure that may be useful to create a SNPs panel in the vitamin D biochemical pathway genes that are known risk factors in cancer and other chronic diseases. As a consequence, this analysis is of great importance to identify the group with the highest risk of incidence of these diseases and thereby contribute to preventive strategies or diagnosis of these illnesses at an early stage.

## Figures and Tables

**Table 1:** Characteristics of vitamin D binding protein (VDBP). VDBP polymorphisms (based on Bouillon 2020 [53])**.**

SNP Locus	GC Name	Codon Variant	Amino Acid Variant
Rs4588	GC1	ACG (Thr->Lys)	Thr-436
Rs4588	GC2	AAG (Thr->Lys)	Lys-436
Rs7041	GC1F	GAT (Asp->Glu)	Asp-432 (416/mature VDBP)
Rs7041	GC1S	GAG (Asp->Glu)	Glu-432 (420/mature VDBP)

**Table 2:** Vitamin D binding protein (VDBP) and human diseases.

Disease	VDBP Influence	Mechanism	Reference
**Cancers**			
Breast cancer	Gc2-2 genotype associated with decreased risk of postmenopausal breast cancer (n = 1402, control: 2608) SNPs: rs17467825, rs2298850 and rs3755967 are associated to the breast cancer risk (n = 818, controls = 935); another study does not support an important role of either calculated circulating free 25(OH)D or circulating VDBP levels in breast cancer risk among predominantly premenopausal women; (controls = 584)	The carcinogenic mechanism is based on the potential to convert Gc to GcMAF, which is a macrophage activator. GcMAF may enhance proapoptotic enzymes activity and induce cell apoptosis via JNK1/2 and p387 pathway—that may inhibit cancer development	[51,54,114,115,116]
Prostate cancer	Decreased risk in of prostate cancer associated with higher serum VDBP levels in men with lower than median 25(OH)D status, where elevated risk in men with higher than median 25(OH)D concentration (n = 950, control = 964); SNP: Rs2282679 in *Gc* has no significant correlation with non-aggressive and aggressive prostate cancer (n = 10,572, controls = 4975)	Extracellular concentrations of VDBP and 25(OH)D result in an upregulation of megalin-mediated internalization of SHBG-bound testosterone	[65,117]
Pancreatic cancer	Higher serum 25(OH)D and serum VDBP are associated with higher pancreatic cancer risk (n = 234, control = 234) among Finnish men population; VDBP or 25(OH)D were not associated with pancreatic cancer (n = 295, two controls n = 590); rs2282679, rs7041 and rs4588 found no significant correlation with pancreas cancer	Reducing free 25(OH)D by VDBP decreases bioavailability; high concentration of VDBP and 25(OH)D could potentially displace 1,25(OH)D with its antitumorigenic properties	[64,70,71]
Lung cancer	VDBP low serum concentration might be a predictor of subsequent death from non-small cell lung cancer (n = 148 lung cancer patients, 68 patients with other intrathoracic tumors and 33 noncancer controls); GC2-1f combination (TT-CA) has significant and protective association with lung cancer (n = 113, control = 113); Rs7041 in GC gene reduces the risk of Non-Small Cell Lung Cancer risk (n = 446, controls = 425)	Conversion of VDBP to GcMAF may be reduced in malignancy due to the action of α-N-acetylogalactosaminidasa and as a result it might lower macrophage activation	[72,75,90]
Colorectal cancer	Rs7041 (TG/GG) significant association with colorectal cancers among age 60 years old and older (n = 282, control = 113); Rs4588 (CA/AA) significant association with cancer in males aged 60 years old or less (n = 282, control = 113); Both: Gc/Rs7041 and CYP2R1/rs10741657 polymorphisms decreases the risk of colorectal cancer about 9–12% (n = 920, controls = 1743)		[74,76]
Basal cell carcinoma	SNP may affect skin carcinogenesis. Among patients with rs7041 and rs4588 233 of them developed BCC and 52.4% among those patients developed multiple BCCs (n = 7983). GC1s homozygotes had lower BCC risk. Rs7041 was associated with BCC development among the youngest group.	SNPs may be associated with BCC development among younger patients	[77]
Cutaneous Melanoma	Association between VDBP rs12512631 and risk of cutaneous melanoma among Spanish population (n = 530, controls = 314); No association between VDBP rs1155563 and rs7041 and melanoma risk or prognosis (n = 305, controls = 370)	VDBP variants may influence on vitamin synthesis and distribution	[78,79,80]
**Other important diseases**			
Thyroid autoimmunity disorders	Intron 8 (TAAA)n-Alu repeat polymorphism correlates with Graves’ disease (n = 561) but no association with Hashimoto’s thyroiditis; VDBP polymorphisms may contribute to development of autoimmune diseases (n = 332, control = 185).	Ability of VDBP to binding; linkage with nearby gene, affecting on immune system by VDBP’s macrophage activating role	[86,87]
Obesity	Possible role of VDBP in the relation between body fat mass and vitamin D metabolism rs17467825 and its corresponding haplotype GAA—strongest association in females; VDBP has an influence on PFM (percentage of fat mass), more significant associations are more female-specific	A lipid-bound VDBP fraction	[47,88,89,118,119,120,121,122]
Diabetes mellitus	People with Gc1S-2 and 1S-1S had higher fasting plasma insulin concentration than 1F-1F; rs7041 (Glu/Glu-416) and rs4588 (Lys/Lys-420) variants of VDBP were higher in type 2 diabetic comparing to control group (n = 104, controls = 107)	Polymorphisms of VDBP might be associated with insulin resistance in Japanese population with normal glucose tolerance. It might contribute to type 2 diabetes development. VDBP affects glucose metabolism by modulating the action of metabolites of vitamin D; Vitamin D stimulates synthesis of insulin, effects on β-cells and protects them against destruction by inflammatory cytokines	[81,82,83,84,123,124,125,126]
Bone metabolism	An inverse correlation between serum VDBP levels and BMD; A highly significant difference in premenopausal bone fracture risk among women with different VDBP phenotypes (VDBP1-1>VDBP2-1>VDBP2-2); SNPs in the VDBP gene might be associated with BMD; VDBP rs4701 is associated with lower BMD-L4 and higher risk of osteoporosis; multiple of the VDBP SNPs might increase the risk of osteoporosis in postmenopausal women	Phenotype of VDBP is mediated by VDBP-MAF and activation osteoclasts	[90,93,127,128,129,130,131,132,133]
Rheumatoid arthritis	correlation between RA and rs2282679 SNP	1,25(OH)2D3 may have inhibitory effect on osteoclasts formation that is induced by IL-22	[95,97]
Ankylosing spondylitis	Patients with G alleles at rs222016 and rs222020 and an allele at rs3733359 show decreased risk of peripheral arthritis; rs4752 polymorphisms are associated with development of uveitis. Haplotype analysis showed that AGGA haplotype protects against peripheral arthritis development in ankylosing spondylitis patients (n = 223, control = 239).		[48]
Asthma	Upregulation of DBP expression in patients with diphenyl-methane disocyanate occupational asthma DBP SNPs rs4588 and rs7041 are associated with the risk of asthma and the DBP1 allele might confer a protective effect; patients with GC2 (compared to GC1) haplotype are more susceptible for the development of asthma; Gc1, Gc2 was significantly associated with the risk of asthma (n = 467, control = 288); Rs7041 and rs4588 associated with increased risk of bronchial asthma (n = 143, controls = 143); Rs4588 CA and AA genotypes had protective effect, while rs7041 GG genotype had significantly higher frequency among patients diagnosed with asthma (n = 96, controls = 96)	VDBP and enhancing the chemotactic activity of monocytes and neutrophils; VDBP modules Th2-mediated inflammation and influences the susceptibility to asthma	[101,102,103,104,105]
Chronic obstructive pulmonary disease	GC1f and GC2 alleles may be linked to sputum hypersecretion in COPD patients; Gc2 protects against COPD (n = 140, control = 480) 1F-1S genotype was protective factor against deficiency of vitamin D among Korean patients (n = 175); High frequencies of the haplotypes in rs7041 and rs4588—GC1S/1S among COPD patients (n = 233)	VDBP has the potential to influence the respiratory function by determining vitamin D bioavailability and via direct effects on innate cell function	[101,106,108,109]
Tuberculosis	VDBP2-2 phenotype strongly associated to susceptibility to TB among Gujarati Asians (n = 534, control = 400); Among Taiwan patients the GC1F carriers were associated with tuberculosis (n = 198, controls = 170)	Reduced ability of VDBP2 to conversion VDBP to VDBP-MAF	[107,134]
Coronary artery diseases	A strong interaction between A allele VDR rs1544410 and G allele of VDBP rs7041 genes in a protective role; strong association between vitamin D deficiency, lipid profile and the VDR rs1544410G>A and rs7T41>G VDBP genes polymorphisms (n = 157, control = 182) No correlation between rs7041, rs4588 and CAD (n = 1080).		[113,135]
